# Supplementary material for: Identification of the Transcription Factor ATF3 as a Direct and Indirect Regulator of the LDLR
Source: Metabolites. 2022 Sep 6;12(9):840. doi: 10.3390/metabo12090840 (PMC9504235; doi:10.3390/metabo12090840)
Supplement: Supplementary file 1 [file metabolites-12-00840-s001.zip › Supplementary file_Members of the DigiMed Bayern Consortium.pdf]

*DigiMedBayernConsortium:

Jonathan Adam, Institut für Medizinische Informationsverarbeitung Biometrie und Epidemiologie, Ludwig-Maximilians-Universität München, Munich, Germany; Institut für Epidemiologie, Helmholtz Zentrum München, Munich, Germany; Reiner Anselm, Institut Technik – Theologie – Naturwissenschaften, Ludwig-Maximilians-Universität München, Munich, Germany; Sara Ates, Deutsches Herzzentrum München, Klinik für Herz- und Kreislauferkrankungen, Technische Universität München, Munich, Germany; Sabine Bauer, Deutsches Herzzentrum München, Klinik für Herz- und Kreislauferkrankungen, Technische Universität München, Munich, Germany; Nicole Beck, Deutsches Herzzentrum München, Klinik für Herz- und Gefäßchirurgie, Technische Universität München, Munich, Germany; Jürgen Beckmann, Fakultät für Sport- und Gesundheitswissenschaften, Technische Universität München, Munich, Germany; Riccardo Berutti, Institut für Neurogenomik, Helmholtz Zentrum München, Munich, Germany; Stefan Brandmaier, Institut für Epidemiologie, Helmholtz Zentrum München, Munich, Germany; Theresa Brunet, Institut für Humangenetik, Technische Universität München, Munich, Germany; Salvatore Cassese, Deutsches Herzzentrum München, Klinik für Herz- und Kreislauferkrankungen, Technische Universität München, Munich, Germany; Diana David-Rus, Deutsches Herzzentrum München, Klinik für Herz- und Kreislauferkrankungen, Technische Universität München, Munich, Germany; Manuela Decker, Deutsches Herzzentrum München, Klinik für Herz- und Kreislauferkrankungen, Technische Universität München, Munich, Germany; Martin Dichgans, Institut für Schlaganfall- und Demenzforschung, Ludwig-Maximilians-Universität München, Munich, Germany; Philine Diesselhorst, Institut Technik – Theologie – Naturwissenschaften, Ludwig-Maximilians-Universität; Horst Domdey, BioM Biotech Cluster Development GmbH, Martinsried, Germany; Martina Dreßen, Deutsches Herzzentrum München, Klinik für Herz- und Gefäßchirurgie, Technische Universität München, Munich, Germany; Arne Dressler, Institut Technik – Theologie – Naturwissenschaften, Ludwig-Maximilians-Universität München, Munich, Germany; Florent Dufour, Leibniz-Rechenzentrum, Munich, Germany; Sven Duscha, Deutsches Herzzentrum München, Klinik für Herz- und Kreislauferkrankungen, Technische Universität München, Munich, Germany; Hans-Henning Eckstein,

Klinik und Poliklinik für Vaskuläre und Endovaskuläre Chirurgie, Klinikums rechts der Isar, , Technische Universität München, Munich, Germany; Aiman Farzeen, Institut für Humangenetik, Technische Universität München, Munich, Germany; Institut für Epidemiologie, Helmholtz Zentrum München, Munich, Germany; Therese Feiler, Institut Technik – Theologie – Naturwissenschaften, Ludwig-Maximilians-Universität München, Munich, Germany; Ines Gall, Deutsches Herzzentrum München, Institut für Laboratoriumsmedizin, Technische Universität München, Munich, Germany; Ulrich M. Gassner, Juristische Fakultät, Universität Augsburg, Augsburg, Germany; Christian Gieger, Institut für Epidemiologie, Helmholtz Zentrum München, Munich, Germany; Monica Gotor-Blazquez, Deutsches Herzzentrum München, Klinik für Herz- und Kreislauferkrankungen, Technische Universität München, Munich, Germany; Ulrich Güldener, Deutsches Herzzentrum München, Klinik für Herz- und Kreislauferkrankungen, Technische Universität München, Munich, Germany; Nicolay Hammer, Max-Planck-Institut für Biochemie, Munich, Germany; Johann Hawe, Deutsches Herzzentrum München, Klinik für Herz- und Kreislauferkrankungen, Technische Universität München, Munich, Germany; Thomas Hendel, Institut für Epidemiologie, Helmholtz Zentrum München, Munich, Germany; Stefan Holdenrieder; Deutsches Herzzentrum München, Institut für Laboratoriumsmedizin, Technische Universität München, Munich, Germany; Stephan Jonas, Institut für Informatik, Technische Universität München, Munich, Germany; Adnan Kastrati, Deutsches Herzzentrum München, Klinik für Herz- und Kreislauferkrankungen, Technische Universität München, Munich, Germany; Wolfgang Kempf, Klinikums rechts der Isar, Technische Universität München, Munich, Germany; Thorsten Keßler, Deutsches Herzzentrum München, Klinik für Herz- und Kreislauferkrankungen, Technische Universität München, Munich, Germany; Wolfgang Koenig, Deutsches Herzzentrum München, Klinik für Herz- und Kreislauferkrankungen, Technische Universität München, Munich, Germany; Florian Kohlmayer, Bitcare, Munich, Germany; Markus Krane, Deutsches Herzzentrum München, Klinik für Herz- und Gefäßchirurgie, Technische Universität München, Munich, Germany; Dieter Kranzlmüller, Institut für Informatik, Ludwig-Maximilians-Universität München, Munich, Germany; Harald Lahm, Deutsches Herzzentrum München, Klinik für Herz- und Gefäßchirurgie, Technische Universität München,

Munich, Germany; Rüdiger Lange, Deutsches Herzzentrum München, Klinik für Herz- und Gefäßchirurgie, Technische Universität München, Munich, Germany; Andreas Lehmann, Bitcare GmbH, Munich, Germany; Ling Li, Deutsches Herzzentrum München, Klinik für Herz- und Kreislauferkrankungen, Technische Universität München, Munich, Germany; Birgit Linkohr, Institut für Epidemiologie, Helmholtz Zentrum München, Munich, Germany; Lars Maegdefessel, Klinik und Poliklinik für Vaskuläre und Endovaskuläre Chirurgie, Klinikums rechts der Isar, Technische Universität München, Munich, Germany; Matthias Mann, Max-Planck-Institut für Biochemie, Munich, Germany; Rainer Malik, Institut für Schlaganfall- und Demenzforschung, Ludwig-Maximilians-Universität München, Munich, Germany; Thomas Meitinger, Institut für Humangenetik, Technische Universität München, Munich, Germany; Irina Neb, Deutsches Herzzentrum München, Klinik für Herz- und Gefäßchirurgie, Technische Universität München, Munich, Germany; Tina O'Hehir, Deutsches Herzzentrum München, Klinik für Herz- und Kreislauferkrankungen, Technische Universität München, Munich, Germany; Shichao Pang, Deutsches Herzzentrum München, Klinik für Herz- und Kreislauferkrankungen, Technische Universität München, Munich, Germany; Benedikt Perl, Fakultät für Sport- und Gesundheitswissenschaften, Technische Universität München, Munich, Germany; Annette Peters, Institut für Medizinische Informationsverarbeitung Biometrie und Epidemiologie Ludwig-Maximilians-Universität München, Munich, Germany; Institut für Epidemiologie, Helmholtz Zentrum München, Munich, Germany; Fatemeh Peymani, Institut für Humangenetik, Technische Universität München, Munich, Germany; Roland Pichler, Max-Planck-Institut für Biochemie, Munich, Germany; Heiko Pfister, Deutsches Herzzentrum München, Institut für Laboratoriumsmedizin, Technische Universität München, Munich, Germany; Paola Pisano, Max-Planck-Institut für Biochemie, Munich, Germany; Holger Prokisch, Institut für Humangenetik, Technische Universität München, Munich, Germany; Lara Marie Reimer, Institut für Informatik, Technische Universität München, Munich, Germany; Michaela Sander, Deutsches Herzzentrum München, Institut für Laboratoriumsmedizin, Technische Universität München, Munich, Germany; Veronika Sanin, Deutsches Herzzentrum München, Klinik für Herz- und Kreislauferkrankungen, Technische Universität München, Munich, Germany; Lea

Dewi Schlieben, Institut für Humangenetik, Technische Universität München, Munich, Germany; Yannick Schlote, Institut Technik – Theologie – Naturwissenschaften, Ludwig-Maximilians-Universität München, Munich, Germany; Sofie Schmid, Klinikums rechts der Isar, Technische Universität München, Munich, Germany; Raphael Schmieder, Deutsches Herzzentrum München, Klinik für Herz- und Kreislauferkrankungen, Technische Universität München, Munich, Germany; Heribert Schunkert, Deutsches Herzzentrum München, Klinik für Herz- und Kreislauferkrankungen, Technische Universität München, Munich, Germany; Megi Sharikadze, Leibniz-Rechenzentrum, Munich, Germany; Ankit Sinha, Max-Planck-Institut für Biochemie, Munich, Germany; Fabian Starnecker, Deutsches Herzzentrum München, Klinik für Herz- und Kreislauferkrankungen, Technische Universität München, Munich, Germany; Medini Steger, Max-Planck-Institut für Biochemie, Munich, Germany; Sophia Steigerwald, Max-Planck-Institut für Biochemie, Munich, Germany; Ruoyu Sun, BioM Biotech Cluster Development GmbH, Martinsried, Germany; Moritz von Scheidt, Deutsches Herzzentrum München, Klinik für Herz- und Kreislauferkrankungen, Technische Universität München, Munich, Germany; Matias Wagner, Institut für Humangenetik, Technische Universität München, Munich, Germany; Annie Westerlund, Deutsches Herzzentrum München, Klinik für Herz- und Kreislauferkrankungen, Technische Universität München, Munich, Germany; Jens Wiehler, BioM Biotech Cluster Development GmbH, Martinsried, Germany; Michael Wierer, Max-Planck-Institut für Biochemie, Munich, Germany; Peter Zinterhof, Leibniz-Rechenzentrum, Munich, Germany;
