# Supplementary figures and images for: Identification of the Transcription Factor ATF3 as a Direct and Indirect Regulator of the LDLR
Source: Metabolites. 2022 Sep 6;12(9):840. doi: 10.3390/metabo12090840 (PMC9504235; doi:10.3390/metabo12090840)

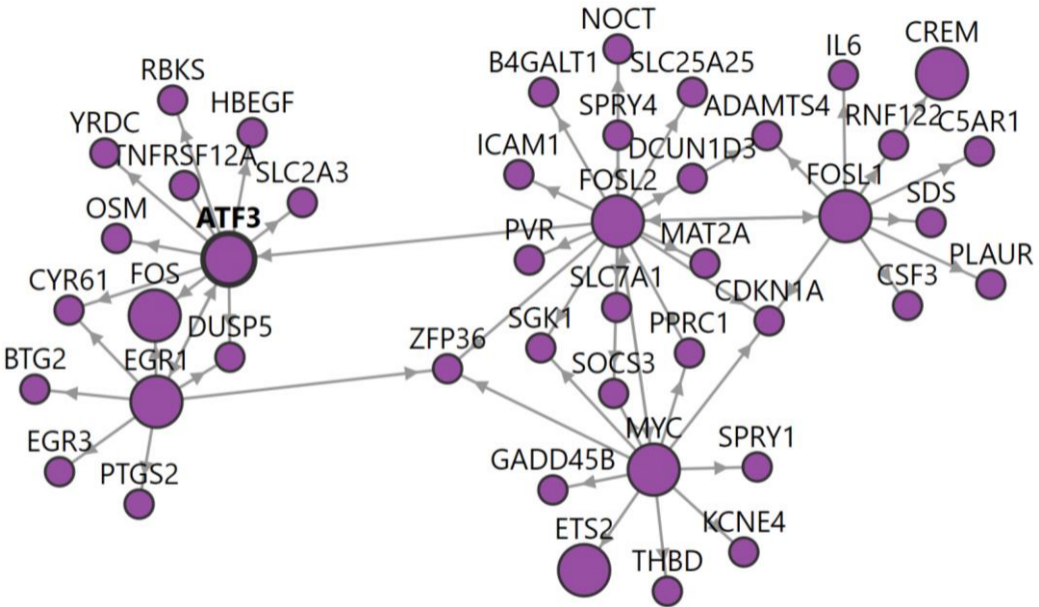

Supplement: Supplementary file 1 [file metabolites-12-00840-s001.zip › Figure S1.pdf]
